# Supplementary material for: Autophagy promotes jasmonate-mediated defense against nematodes
Source: Nat Commun. 2023 Aug 8;14:4769. doi: 10.1038/s41467-023-40472-x (PMC10409745; doi:10.1038/s41467-023-40472-x)
Supplement: Supplementary file 1 — Supplementary Information [file 41467_2023_40472_MOESM1_ESM.pdf]

Autophagy promotes jasmonate-mediated defense against nematodes

Jinping Zou, Xinlin Chen, Chenxu Liu, Mingyue Guo, Mukesh Kumar Kanwar, Zhenyu Qi, Ping Yang, Guanghui Wang, Yan Bao, Diane C Bassham, Jingquan Yu and Jie Zhou

Supplementary Table 1. Target sequences used for CRISPR-Cas9 technology

Name of sgRNA	Sequence
ATG4-sgRNA	TGCTCGCATCCTCAAGTATC
ATG7-sgRNA	TAACCTCCTGCAAGCCGGCA
JAM1-sgRNA	CAAACGCCGCTAATTTTAGC
JAM1/2-sgRNA	ACTGGGAATATGTTGTGGAG
JAM2-sgRNA	GATGGGGAGATGGTTGTTGT
ERF1-sgRNA	TCCGAAGAGATGCTTCTCTT
MED25-sgRNA1	ATAGTGCTTGCCTGGTTCAG
MED25-sgRNA2	ACATGGATACCTTTTTGCAG

Supplementary Table 2. Primers used for plasmid construction

Name	Sequence
JAM1-HA-F	ttacaattaccatggggcgcgccATGGTTACTGGGAATATGTTGTGG
JAM1-HA-R	aacatcgtatgggtaggtaccTTGCCCTACCGGTGAAAGTTG
ERF1-GFP-F	ctctcgagctttcgcgagctcATGGATTCTTCTTCTTCTTCATCTCA
ERF1-GFP-R	gcccttgctcaccatggatccCCATGGACTAAAATAAGTTGCATCA
JAM1-GFP-F	ctctcgagctttcgcgagctcATGGTTACTGGGAATATGTTGTGG
JAM1-GFP-R	gcccttgctcaccatggatccTTGCCCTACCGGTGAAAGTTG
JAM2-GFP-F	ctctcgagctttcgcgagctcATGACTATGTTGTGGAGTGATGAGG
JAM2-GFP-R	gcccttgctcaccatggatccGAGAGAGTTTGATTCACCAGCAAA
JAM3-GFP-F	ctctcgagctttcgcgagctcATGGCAGAGAAATTTTTTCTGAAGG
JAM3-GFP-R	gcccttgctcaccatggatccTTTAGACAGAGCAGCTGTCAGCTT
JAM1^{mAIM1}- GFP-F	ATCgcctggcaagccTCGCGGTCTAAGTCAGGGG
JAM1^{mAIM1}- GFP-R	CGAggcttgccaggcGATGGCATAATTCCAGCTAAAATTAG
pGADT7-JAM1-F	gccatggaggccagtgaattcATGGTTACTGGGAATATGTTGTGG
pGADT7-JAM1-R	cagctcgagctcgatggatccTTGCCCTACCGGTGAAAGTTG
pGADT7-JAM2-F	gccatggaggccagtgaattcATGACTATGTTGTGGAGTGATGAGG
pGADT7-JAM2-R	cagctcgagctcgatggatccGAGAGAGTTTGATTCACCAGCAAA
pGADT7-JAM3-F	gccatggaggccagtgaattcATGGCAGAGAAATTTTTTCTGAAGG
pGADT7-JAM3-R	cagctcgagctcgatggatccTTTAGACAGAGCAGCTGTCAGCTT
pGADT7-JAM1^{m}AIM1-F	ATCgcctggcaagccTCGCGGTCTAAGTCAGGGG
pGADT7-JAM1mAIM1-R	CGAggcttgccaggcGATGGCATAATTCCAGCTAAAATTAG
pGADT7-JAM2mAIM1-F	AATTgcctggcaagccTCGCGGTCTAAGTTGGGGG
pGADT7-JAM2mAIM1-R	GAggcttgccaggcAATTGCATAATTCCAACTAAAATTACTTG
pGADT7-JAM3mAIM1-F	ATCgcctggcaagccGCAAAGTCGAAATCTGGAAAATC
pGADT7-JAM3mAIM1-R	TGCggcttgccaggcGATTGCATAAGTCCAATCAGACCC
pGADT7-JAM1mAIM2-F	gcctatgccgcccCGAGCTGTTGTTCCAAATATCTCC
pGADT7-JAM1mAIM2-R	AGCTCGggcggcataggcCCGCTGGTTCAACTTTTCCC
pGADT7-JAM2mAIM2-F	gcctatgcagcccCGAGCTGTTGTTCCAAATATTTCC
pGADT7-JAM2mAIM2-R	AGCTCGggctgcataggcCCGCTGGTTCAGCTTCTCC
pGADT7-JAM3mAIM2-F	AGGgcctatgctgccAGAGCAGTTGTTCCGAATATCTCA
pGADT7-JAM3mAIM2-R	TCTggcagcataggcCCTCTGGTTTAGCTTCTCACGC
pGADT7-ERF1-F	gccatggaggccagtgaattcATGGATTCTTCTTCTTCTTCATCTCA
pGADT7-ERF1-R	cagctcgagctcgatggatccCCATGGACTAAAATAAGTTGCATCA
pGBKT7-JAM1-F	atggccatggaggccgaattcATGGTTACTGGGAATATGTTGTGG
pGBKT7-JAM1-R	ccgctgcaggtcgacggatccTTGCCCTACCGGTGAAAGTTG
pGBKT7-JAM2-F	atggccatggaggccgaattcATGACTATGTTGTGGAGTGATGAGG
pGBKT7-JAM2-R	ccgctgcaggtcgacggatccGAGAGAGTTTGATTCACCAGCAAA
pGBKT7-JAM3-F	atggccatggaggccgaattcATGGCAGAGAAATTTTTTCTGAAGG
pGBKT7-JAM3-R	ccgctgcaggtcgacggatccTTTAGACAGAGCAGCTGTCAGCTT
pGBKT7-ATG8a-F	atggccatggaggccgaattcATGGCCAAAAGCTCCTTCAAA
pGBKT7-ATG8a-R	ccgctgcaggtcgacggatccGAAGGATCCGAAGGTATTCTCGC
pGBKT7-ATG8b-F	atggccatggaggccgaattcATGGCCAAAAGTTCTTTCAAGC
pGBKT7-ATG8b-R	ccgctgcaggtcgacggatccATTTCCAAGCTCAACGAACCC
pGBKT7-ATG8c-F	atggccatggaggccgaattcATGTTCTTTAGATGTTTGAAATTTGCA
pGBKT7-ATG8c-R	ccgctgcaggtcgacggatccCTTCTTCTTATCAATGTCGGAGATATCA
pGBKT7-ATG8d-F	atggccatggaggccgaattcATGGACCATTCTTGTTATTTTCCTT
pGBKT7-ATG8d-R	ccgctgcaggtcgacggatccAAAGGATCCAAATGTATTCTCTCCA
pGBKT7-ATG8e-F	atggccatggaggccgaattcATGATAAAAAAAAATTAGGCTACATACATTATT
pGBKT7-ATG8e-R	ccgctgcaggtcgacggatccTGTTCTTGCTTGAACAAACTCTTTG
pGBKT7-ATG8f-F	atggccatggaggccgaattcATGGCTAAGAGCTCATTCAAGCA
pGBKT7-ATG8f-R	ccgctgcaggtcgacggatccCAGTTCGCTCAGGACCCCG
pGBKT7-ATG8h-F	atggccatggaggccgaattcATGGGGAAGACCTTCAAAGATGA
pGBKT7-ATG8h-R	ccgctgcaggtcgacggatccAGAGTGACCACCAAAGGTTTTCTC

Supplementary Table 2 continued

pGBKT7-MED25-F	atggccatggaggccgaattcATGGTGGACAAACTGATCGTCG
pGBKT7-MED25-R	ccgctgcaggtcgacggatccATTCATAAACCCGCCTCCTGG
pbridge-MED25-mcs1-F	ttgactgtatcgccggaattcATGGTGGACAAACTGATCGTCG
pbridge-MED25-mcs1-R	tggctgcaggtcgacggatccATTCATAAACCCGCCTCCTGG
pBridge-JAM1-F	aagaagagaaaggtggcggccgcATGGTTACTGGGAATATGTTGTGG
pBridge-JAM1-R	gggagatcagcccgaagatctTTGCCCTACCGGTGAAAGTTG
JAM1-nYFP-F	atttacgaacgatagttaattaaATGGTTACTGGGAATATGTTGTGG
JAM1-nYFP-R	actgccacctcctccactagtTTGCCCTACCGGTGAAAGTTG
JAM2-nYFP-F	atttacgaacgatagttaattaaATGACTATGTTGTGGAGTGATGAGG
JAM2-nYFP-R	actgccacctcctccactagtGAGAGAGTTTGATTCACCAGCAAA
JAM3-nYFP-F	atttacgaacgatagttaattaaATGGCAGAGAAATTTTTTCTGAAGG
JAM3-nYFP-R	actgccacctcctccactagtTTTAGACAGAGCAGCTGTCAGCTT
JAM1^{mAIM1}-nYFP-F	ATCgcctggcaagccTCGCGGTCTAAGTCAGGGG
JAM1^{mAIM1}-nYFP-R	CGAggcttgccaggcGATGGCATAATTCCAGCTAAAATTAG
JAM2^{mAIM1}-nYFP-F	AATTgcctggcaagccTCGCGGTCTAAGTTGGGGG
JAM2^{mAIM1}-nYFP-R	GAggcttgccaggcAATTGCATAATTCCAACTAAAATTACTTG
JAM3^{mAIM1}-nYFP-F	ATCgcctggcaagccGCAAAGTCGAAATCTGGAAAATC
JAM3^{mAIM1}-nYFP-R	TGCggcttgccaggcGATTGCATAAGTCCAATCAGACCC
cYFP-ATG8a-F	tcggcatggacgagctgtacaAGATGGCCAAAAGCTCCTTCA
cYFP-ATG8a-R	ccagtgaattcccggggatccTCAGAAGGATCCGAAGGTATTCTC
ERF1-cYFP-F	atttacgaacgatagttaattaacATGGATTCTTCTTCTTCTTCATCTCA
ERF1-cYFP-R	actgccacctcctccactagtCCATGGACTAAAATAAGTTGCATCA
MED25-cYFP-F	atttacgaacgatagttaattaacATGGTGGACAAACTGATCGTCG
MED25-cYFP-R	actgccacctcctccactagtATTCATAAACCCGCCTCCTGG
MED25-nYFP-F	atttacgaacgatagttaattaacATGGTGGACAAACTGATCGTCG
MED25-nYFP-R	actgccacctcctccactagtATTCATAAACCCGCCTCCTGG
MYC-ATG8a-F	catatggggctgcaggaattcATGGCCAAAAGCTCCTTCAAA
MYC-ATG8a-R	gggactagaactagtggatccGAAGGATCCGAAGGTATTCTCGC
PET28a-ERF1-F	cagcaaatgggtcgcggatccATGGATTCTTCTTCTTCTTCATCTCA
PET28a-ERF1-R	ctcgagtgcggccgcaagcttCCATGGACTAAAATAAGTTGCATCA
pMAL-MED25-F	gagggaaggatttcagaattcATGGTGGACAAACTGATCGTCG
pMAL-MED25-R	caggtcgactctagaggatccATTCATAAACCCGCCTCCTGG
PET32a-JAM1-F	gccatggctgatatcggatccATGGTTACTGGGAATATGTTGTGG
PET32a-JAM1-R	gcaagcttgtcgacggagctcTTGCCCTACCGGTGAAAGTTG
GST-ERF1-F	gatctggttccgcgtggatccATGGATTCTTCTTCTTCTTCATCTCA
GST-ERF1-R	gtcacgatgcggccgctcgagCCATGGACTAAAATAAGTTGCATCA
GST-JAM1-F	gatctggttccgcgtggatccATGGTTACTGGGAATATGTTGTGG
GST-JAM1-R	ctcgagtcgacccgggaattcTTGCCCTACCGGTGAAAGTTG
SK-ERF1-F	cgctctagaactagtggatccATGGATTCTTCTTCTTCTTCATCTCA
SK-ERF1-R	gataagcttgatatcgaattcCCATGGACTAAAATAAGTTGCATCA
p*ERF1*-LUC-F	gtcgacggtatcgataagcttAAACATTTGTTGATTTGTTATTATATACCTAA
p*ERF1*-LUC-R	cgctctagaactagtggatccCTTTATTCATCAGCCTTGTCTAGTTCG
p*ATG8a*-LUC-F	gtcgacggtatcgataagcttCAGTCTTAATCTTCCATGAAGTTTTGA
p*ATG8a*-LUC-R	cgctctagaactagtggatccATAGATGCAAAAAAAATCGTCTCG
p*ATG8d*-LUC-F	gtcgacggtatcgataagcttTTATTAGACAAACAAAAAAAGACCATAAGA
p*ATG8d*-LUC-R	cgctctagaactagtggatccGTGAGCTAGTCTTAGTAGCTGCCCT
p*ATG13b*-LUC-F	gtcgacggtatcgataagcttCATATGTTCACTTGCCTTTGCAA
p*ATG13b*-LUC-R	cgctctagaactagtggatccTTTCAAAGTATTTGTAATTAAAGTTTAGGTAA

Supplementary Table 3. Primers used for RT-qPCR

Name	Sequence
ATG1a-F	AGTTCGGAAAGTCCCTCATC
ATG1a-R	ATGATAGCAGAGGCAGAAC
ATG1b-F	GGAAAGTCCCTCTT CTGCTC
ATG1b-R	ACTCAATTCTGGGTATGCCA
ATG2-F	GTCATTGAAGAGGCACTGCT
ATG2-R	AGCTAAATCAACACGGCAAG
ATG3-F	GAGAGGAGTTGAACCCGAAG
ATG3-R	CGAAGGAAGTTGACAGCAAA
ATG4-F	AATTGATCCCTCCTTGGCTA
ATG4-R	GATGTGGCAGAGCTACGAGT
ATG5-F	TCAGATGGTGCTGAGATCAAG
ATG5-R	ATTGTTTACCACCCATGCAA
ATG6-F	CCCATGCAGTCAAACAATTC
ATG6-R	CCCTCATGCATTCAAGACAC
ATG7-F	ATTCAACGGCTAACCGTACC
ATG7-R	CAAACTCAGCTTTGGCACAT
ATG8a-F	ACCGGTGATTGTTGAGAAGG
ATG8a-R	GCGCTGAGCTTAATCCTCTT
ATG8b-F	GGAGAGGAGGCAGTCAGAAT
ATG8b-R	AGTCAAATCAGCTGGGACAA
ATG8c-F	TTGGCCAATTTGTTTACGTT
ATG8c-R	AAAGGAATCCGTCTTCATCC
ATG8d-F	AATATCCTTCCTCCCACAGC
ATG8d-R	GCAGAGAGGTTTGACTGCAT
ATG8e-F	TTCGTCGACAATGTCCTACC
ATG8e-R	AAAGGAAACCGTCTTCATCC
ATG8f-F	GGCAATCATGTCTGCAATCT
ATG8f-R	TCAAAGCTACAGTTCGCTCAG
ATG8h-F	CGTGTTTGTGAATAACACCTTG
ATG8h-R	AGCACATGTAGAGGAACCCA
ATG9-F	ATGTGCATCCTGAAATCGAA
ATG9-R	GCCTCTCGAAGAACAAGTCC
ATG10-F	GGAGAACCCTTGGCAATAGA
ATG10-R	TAGTCCCACATGGATGCAAT
ATG12-F	GTGTATGTCAACAGCGCCTT
ATG12-R	AAACAACCAGGAGTTCTCAGC
ATG13b-F	CTGTAGGTGCCCTTGTTCAC
ATG13b-R	AAGCTTTGAGCTCCTCCAAT
ATG18a-F	CAGCGAGTTCACCACTATCC
ATG18a-R	TCCATCCAAGCCAAGAATTA
ATG18b-F	TTGAGGAGACAGCAACACCT
ATG18b-R	TGTTCTGATGGTTGACGTTG
ATG18c-F	GCTTTGCGCTTACAAATGAT
ATG18c-R	CTGCTCTATCTGCGCCTCT
ATG18f-F	TCCGAAGCAGAACTCCAAAT
ATG18f-R	AACCTCAGCCTCTCCACGAC
PDF1.2a-F	ATTTGCAAAGCACCAAGCCAAAC
PDF1.2a-R	CATCATAATCTCTTCTTCAAGCA
PDF1.2b-F	ACTTATGGTCTTGGCAATGGTGCT
PDF1.2b-R	AGTTTGCTACAATGTCCACCTGTA

Supplementary Table 3 continued

JAM1-F	AGCTCTATGCTCCTCCAACC
JAM1-R	GGTTGCTCCGGTGAAATCAA
JAM2-F	GAGGAAGCACCTCTGTGGAT
JAM2-R	CGCTGACCTGTGTCTCTTTG
JAM3-F	GGTTGTGGAGCTTGGTTCTG
JAM3-R	CTTAGCGCCACCTAGACTGA
ERF1-F	CCGAAACAGTCACATCGCAT
ERF1-R	ACACCTCGGTACGACTTCTC
Ubiquitin3-F	GCCGACTACAACATCCAGAAGG
Ubiquitin3-R	TGCAACACAGCGAGCTTAACC
Actin-F	TGGTCGGAATGGGACAGAAG
Actin-R	CTCAGTCAGGAGAACAGGGT

Supplementary Table 4. Primers used for EMSA

Name	Sequence
PDF1.2a-probe-F	CTTAAAATATTTAACAGAAATCCGACCCAGTCAGGGTCAC
PDF1.2a-probe-R	GTGACCCTGACTGGGTCGGATTTCTGTTAAATATTTTAAG
Mu-PDF1.2a-probe-F	CTTAAAATATTTAACAGAAATAAAAAACAGTCAGGGTCAC
Mu-PDF1.2a-probe-R	GTGACCCTGACTGTTTTTTATTTCTGTTAAATATTTTAAG
PDF1.2b-probe-F	ACTTCAGCATGATAGCACTCACCGACAAAGATCATGGCAT
PDF1.2b-probe-R	ATGCCATGATCTTTGTCGGTGAGTGCTATCATGCTGAAGT
Mu-PDF1.2b-probe-F	ACTTCAGCATGATAGCACTCAAAAAAAAAGATCATGGCAT
Mu-PDF1.2b-probe-R	ATGCCATGATCTTTTTTTTTGAGTGCTATCATGCTGAAGT
ATG1a-probe-F	TGAGACTAGCGTGATAACCGACTACACCACCCAGACTTCG
ATG1a-probe-R	CGAAGTCTGGGTGGTGTAGTCGGTTATCACGCTAGTCTCA
ATG1b-probe-F	GTGTGTGAGACTAGCGTGATAACCGACTACACCACCCAGA
ATG1b-probe-R	TCTGGGTGGTGTAGTCGGTTATCACGCTAGTCTCACACAC
ATG8a-probe-F	ATTGTTTTCCCGACCAAATAAGGCAGCCAGGCAGGAATTA
ATG8a-probe-R	TAATTCCTGCCTGGCTGCCTTATTTGGTCGGGAAAACAAT
Mu-ATG8a-probe-F	ATTGTTTTCAAAAAAAAATAAGGCAGCCAGGCAGGAATTA
Mu-ATG8a-probe-R	TAATTCCTGCCTGGCTGCCTTATTTTTTTTTGAAAACAAT
ATG8d-probe-F	TCACGTTACATATGACCGACATAAAGATAAGTAAAAACAA
ATG8d-probe-R	TTGTTTTTACTTATCTTTATGTCGGTCATATGTAACGTGA
Mu-ATG8d-probe-F	TCACGTTACATATGAAAAAAATAAAGATAAGTAAAAACAA
Mu-ATG8d-probe-R	TTGTTTTTACTTATCTTTATTTTTTTCATATGTAACGTGA
ATG13b-probe-F	TAGGGAACATTAAACCGACTCTTGGGGATTTGAATCTATT
ATG13b-probe-R	AATAGATTCAAATCCCCAAGAGTCGGTTTAATGTTCCCTA
Mu-ATG13b-probe-F	TAGGGAACATTAAAAAAAATCTTGGGGATTTGAATCTATT
Mu-ATG13b-probe-R	AATAGATTCAAATCCCCAAGATTTTTTTTAATGTTCCCTA
ERF1-probe-F	TGACGTCATTAAATCCGCCGCCTTGATTGATTTGCCCACG
ERF1-probe-R	CGTGGGCAAATCAATCAAGGCGGCGGATTTAATGACGTCA
Mu-ERF1-probe-F	TGACGTCATTAAATCCAAAAAATTGATTGATTTGCCCACG
Mu-ERF1-probe-R	CGTGGGCAAATCAATCAATTTTTTGGATTTAATGACGTCA

Supplementary Table 5. Primers used for ChIP-qPCR

Name	Sequence
PDF1.2a-ChIP-F	TCTTTTACCCTAATTAGCATAT
PDF1.2a-ChIP-R	TTCTGAAATTTGGGTACTGAAA
PDF1.2b-ChIP-F	TCAACAATCGAACAAGTCAT
PDF1.2b-ChIP-R	CGACGAGTATTACTATGTCG
ERF1-ChIP-F	GTTATTATATACCTAAAACAAAAT
ERF1-ChIP-R	AAAAATAATAATAGGGGACTAGGG
ATG8a-ChIP-F	CTTAATCTTCCATGAAGTTTTGAT
ATG8a-ChIP-R	ATGTGAAGAAGTACAGAACAGTCA
ATG8d-ChIP-F	CATATCTTGGACACCCTTAACATA
ATG8d-ChIP-R	TAGTCTTAGTAGCTGCCCTAATA
ATG13b-ChIP-F	ACTATTCTGTCTTTAATTAATATCAA
ATG13b-ChIP-R	GGTAATCAAAATTTTGAGAAGTA

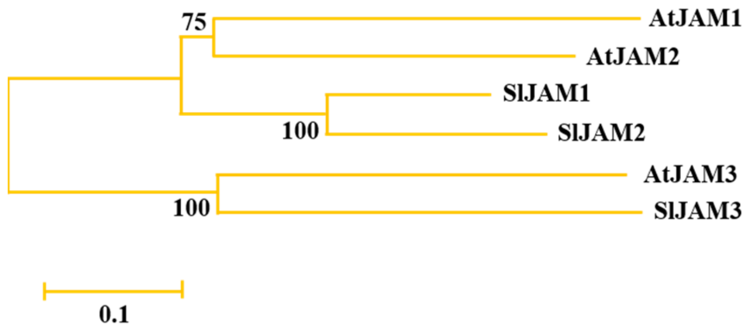

Supplementary Fig. 1 Phylogenetic analysis of JAM homologues in tomato and Arabidopsis. Homologues of JAM proteins are identified using the Sol genomics network (http://solgenomics.net/) and TAIR (www.arabidopsis.org) for tomato and Arabidopsis. Together with three homologues in Arabidopsis and tomato, a phylogenetic tree was built based on their amino acid sequences using the maximum-likelihood method in MEGA5.0 software.

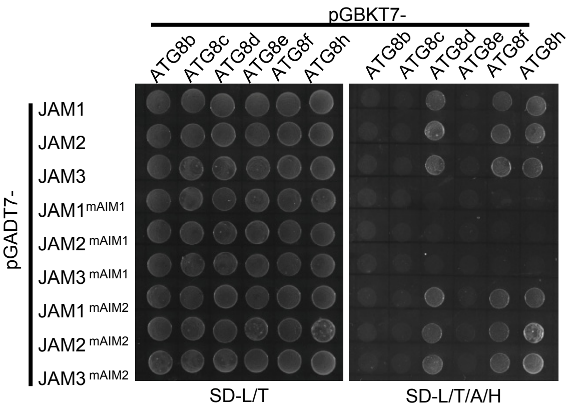

Supplementary Fig. 2 Yeast two-hybrid assays of the interaction between ATG8s (ATG8b-h), and JAM1/2/3, JAM1/2/3^{mAIM1} or JAM1/2/3^{mAIM2}. Protein-protein interactions were evaluated by the different concentration of yeast cells growth on selective media lacking Leu (L), Trp (T), Ade (A), and His (H) (SD-L/T/A/H). All experiments were repeated twice with the similar results.

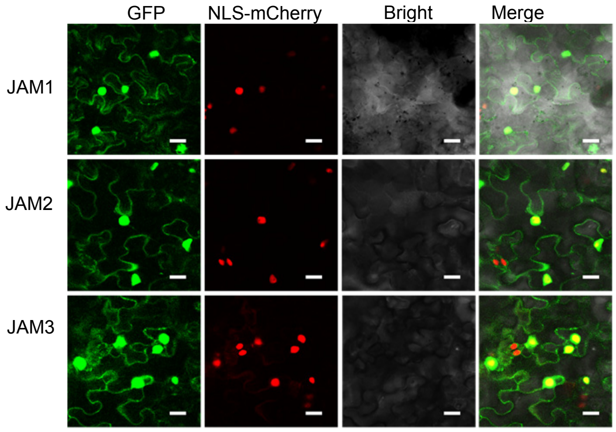

Supplementary Fig. 3 Subcellular localization of tomato JAMs. The left panel displays the JAMs (JAM1, JAM2 and JAM3)-GFP fluorescence whilst the second panel displays the NLS-mCherry fluorescence (nucleus marker). The right panel displays the transmitted light image (Bright filed) overlayed with the JAMs (JAM1, JAM2 and JAM3)-GFP and NLS-mCherry fluorescence. The signals were observed by confocal microscopy 48 h after infiltration and the experiments were repeated twice with the similar results. Bars, 50 μm.

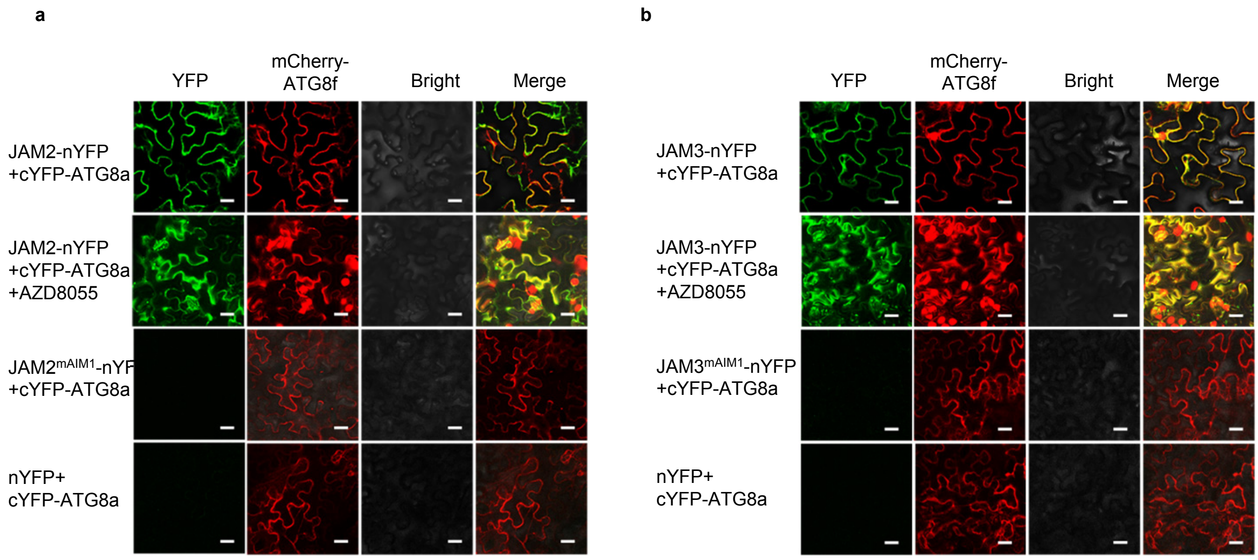

Supplementary Fig. 4 Bimolecular fluorescence complementation assays of the interaction between ATG8a and JAM2, JAM2^{mAIM1} (a) or JAM3, JAM3^{mAIM1} (b). mCherry-ATG8f acts as a marker for autophagosomes. The YFP and mCherry signals were observed by confocal microscopy 48 h after infiltration, then treated with AZD8055 for 3 hours to detect the formation of autophagosomes, and the experiments were repeated twice with the similar results. AZD8055, autophagy activator. Bars, 50 μm.

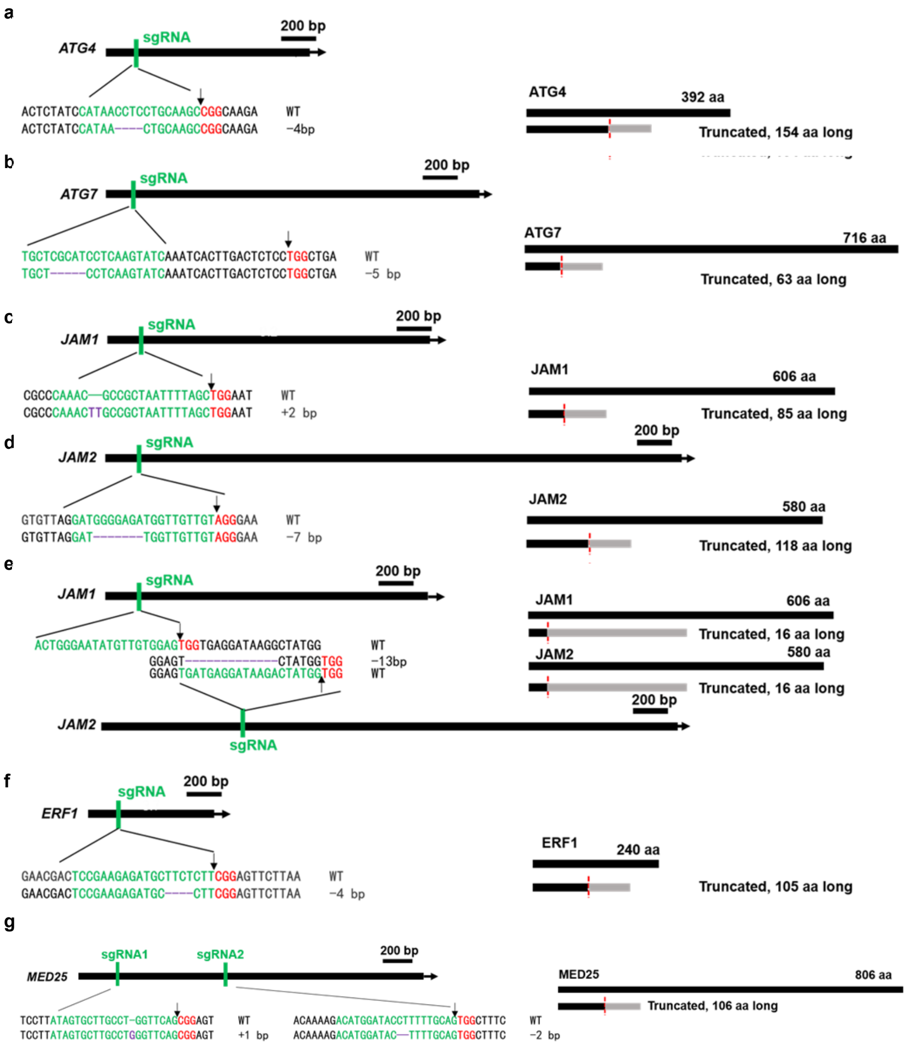

Supplementary Fig. 5 Generation of *atg4* (a), *atg7* (b), *jam1* (c), *jam2* (d), *jam1/2* (e), *erf1* (f) and *med25* (g) mutants using CRISPR/Cas9. Single Guide RNAs (sgRNAs) are indicated in green, protospacer-adjacent motifs (PAMs) are indicated in red and Cas9 cutting sites are indicated by black arrows. Nucleotide deletions or insertions are shown in purple. The predicted mutated proteins are schematically illustrated (right panel) with the frame-shifts indicated by red dashed lines. aa, amino acids. WT; wild-type sequences.

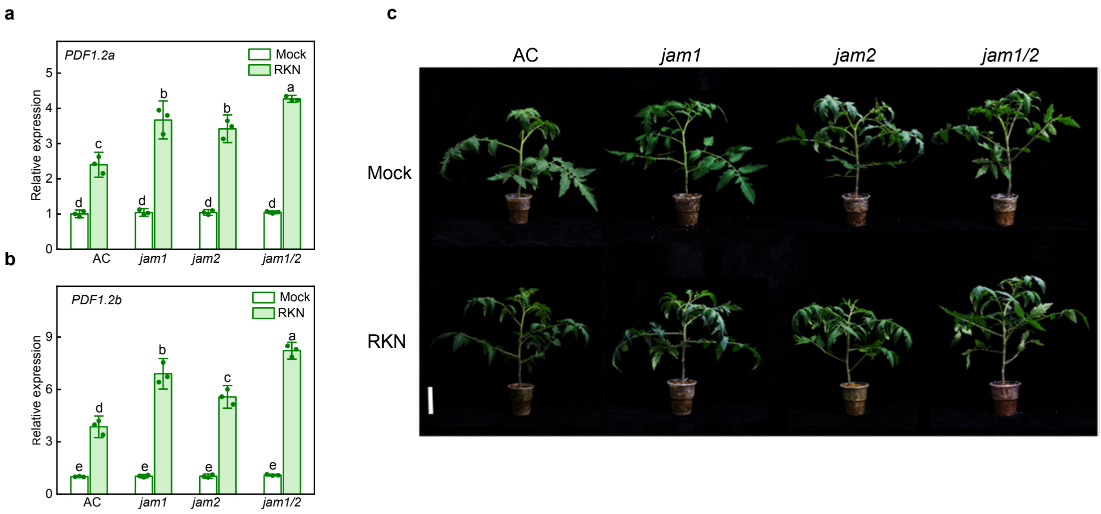

Supplementary Fig. 6 JAM1 and JAM2 negatively regulate tomato resistance to RKNs. a, b, RT-qPCR results showing the transcription levels of *PDF1.2a* (**a**) and *PDF1.2b* (**b**) in Ailsa Craig (AC), *jam1*, *jam2* and *jam1/2* double mutants with or without RKN infection. Total RNA was isolated from root samples collected under control condition or at 48 hpi with RKNs. Transcript levels were determined using RT-qPCR. Error bars represent SD, data represent the mean ± SD (n = 3 biological replicates, individual dots). Different letters above bars indicate a significant difference at the $P < 0.05$ level by one-way ANOVA with Tukey' s multiple comparisions test. Exact *P*-values of statistic tests are provided in the Source data file. **c,** Growth phenotypes of AC, *jam1*, *jam2* and *jam1/2* mutants with or without RKN infection. An average of 25 plants were treated for each genotype, and pictures of each representative lines were shown. The experiments were repeated twice with the similar results. Bar, 8 cm.

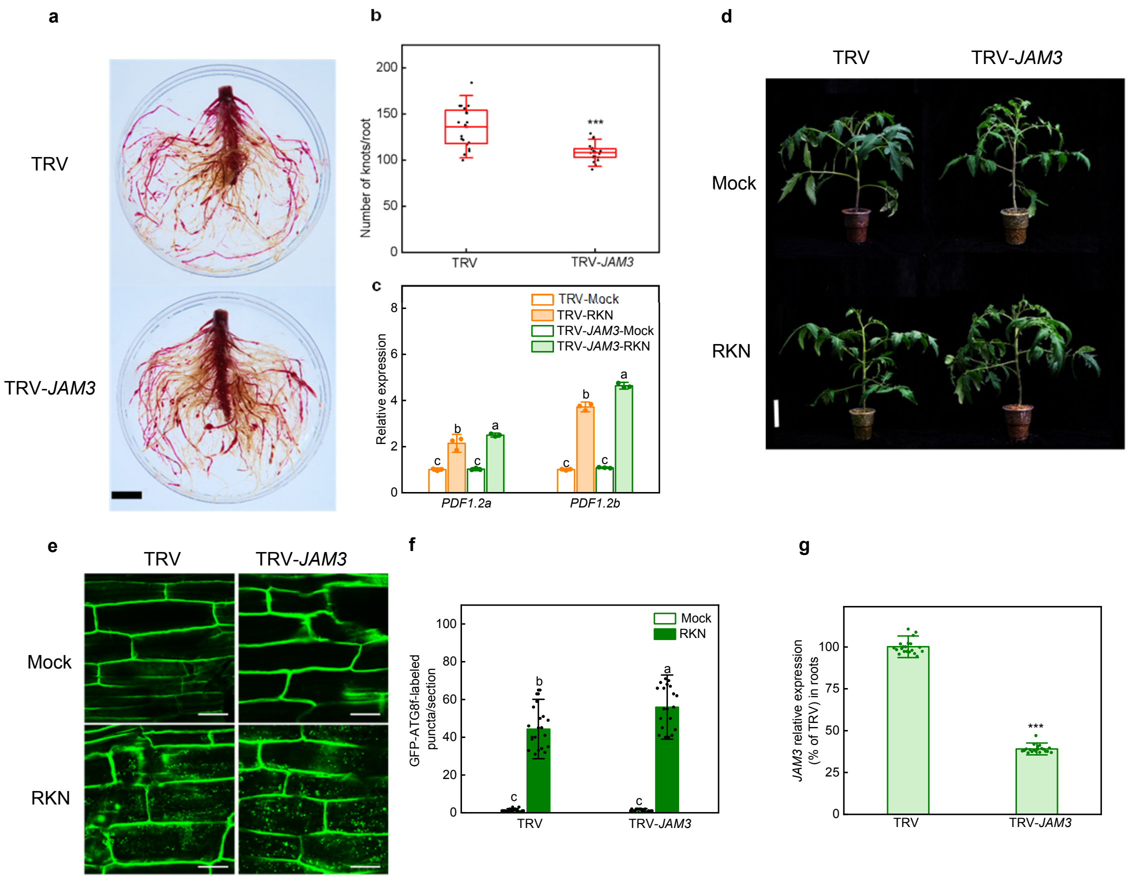

Supplementary Fig. 7 The role of JAM3 in tomato resistance to RKNs. **a**, **b**, Evaluation of resistance of TRV control and *JAM3*-silenced seedlings to RKNs. **a,** Phenotype of RKN reproduction in TRV and *JAM3*-silenced seedlings using acid fuchsin staining after RKN infection. Bar, 1 cm. **b,** The number of root knots of plants at 5 weeks after infection with RKNs. Data are presented as boxplots with each dot representing the datapoint of one biological replicate (n = 20 plants). For the boxplots, the central line indicates the mean value, the bounds of the box show the 1st and 3rd quartile and the whiskers indicate 1.5× interquartile range between the 1st and 3rd quartile. The experiments were repeated twice with the similar results. **c**, RT-qPCR analysis of *PDF1.2a* and *PDF1.2b* in the roots of TRV and TRV-*JAM3* plants with or without RKN infection. Transcript levels were determined using RT-qPCR. Error bars represent SD, data represent the mean ± SD (n = 3 biological replicates, individual dots). **d,** Growth phenotypes of *JAM3*-silenced seedlings in AC plants with or without RKN infection. An average of 25 plants were treated for each genotype, and pictures of each representative lines were shown. Bar, 8 cm. **e,** The direct fluorescence of GFP-ATG8f was detected in the roots with or without RKN infection. Bars, 25 μm. **f,** Quantification of **e**. The number of autophagosomes per image was quantified to calculate the autophagic activity relative to TRV control plants, which was set to 1. Error bars represent SD, data represent the mean ± SD (n = 20 samples, individual dots). The experiments were repeated twice with the similar results. **g**, Silencing efficiency of *JAM3* in Alisa Craig (AC). The levels were presented as percentages compared with that of the control TRV plants, which were defined as 100%. Error bars represent SD, data represent the mean ± SD (n = 20 plants, individual dots). The asterisks in **b** and **g** indicate significant difference as assessed by two sided Student's *t*-tests; *** $P < 0.001$. Different letters above bars indicate a significant difference at the $P < 0.05$ level by one-way ANOVA with Tukey' s multiple comparisions test (**c** and **f**). Exact *P*-values of statistic tests are provided in the Source data file.

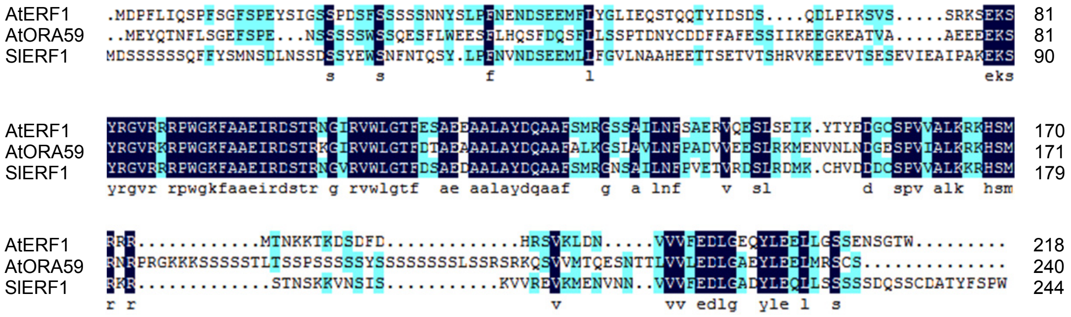

Supplementary Fig. 8 Protein sequence comparison of AtERF1, AtORA59 and SlERF1. The alignment was generated using DNAMAN 8.0 software. The conserved amino acids are highlighted in different colors. The light blue and dark blue boxes indicate homology levels >=75% and =100%, respectively.

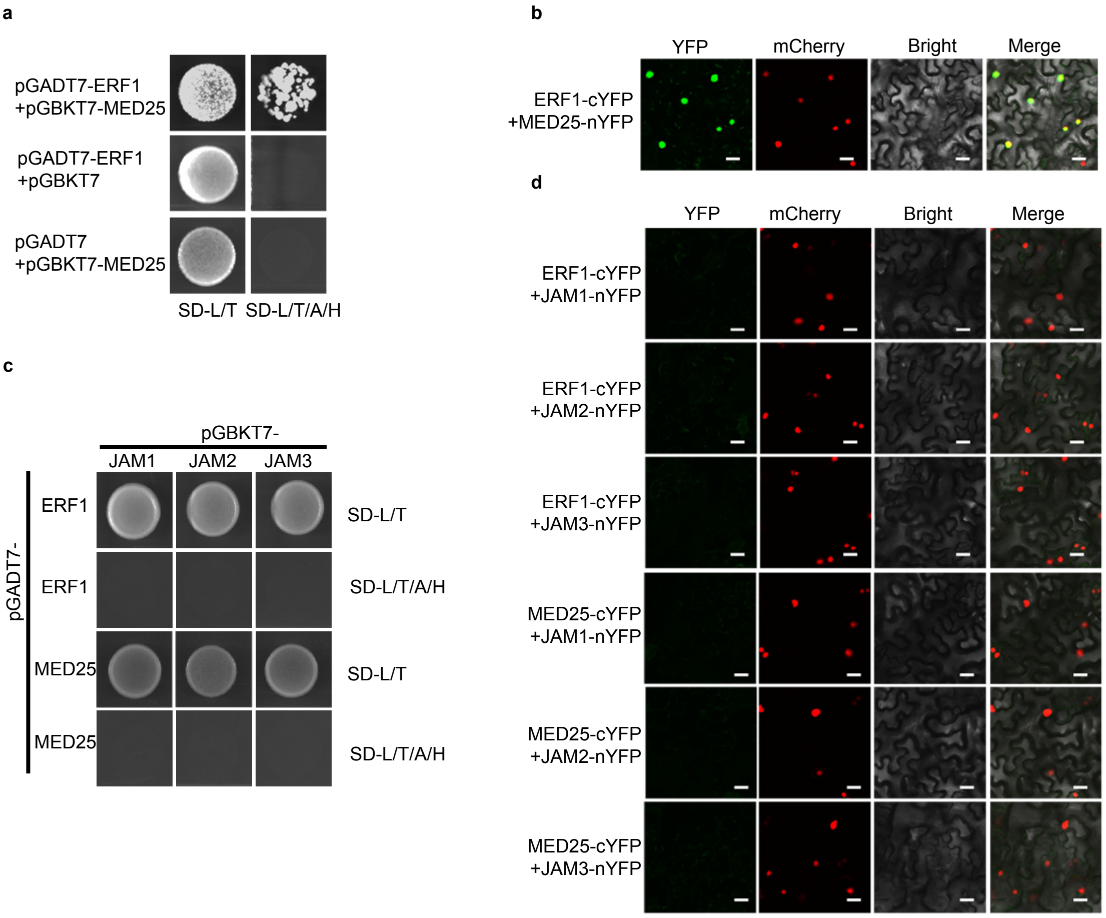

Supplementary Fig. 9 Relationship between ERF1, MED25 and JAM1/2/3. a, c Yeast two-hybrid assays of the interaction between ERF1, MED25 and JAMs. Protein-protein interactions were evaluated by the different concentration of yeast cells growth on selective media lacking lacking Leu (L), Trp (T), Ade (A), and His (H) (SD-L/T/A/H). **b**, **d** Bimolecular fluorescence complementation assays of the interaction between ERF1, MED25 and JAMs. The YFP and mCherry signals were visualized under confocal microscopy 48 h after infiltration. Bars, 50 μm. All the experiments were repeated twice with the similar results.

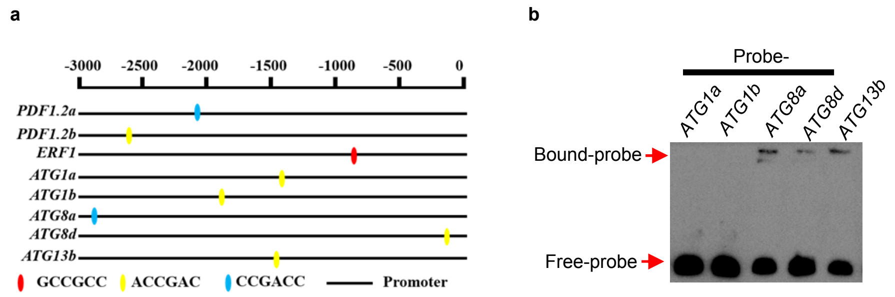

Supplementary Fig. 10 ERF1 binds to the promoters of *ATGs* in vitro. a, ERF binding motifs in the promoters of *PDF1.2a*, *PDF1.2b*, *ERF1*, *ATG1a*, *ATG1b*, *ATG8a*, *ATG8d* and *ATG13b* in tomato. **b**, Electrophoretic mobility shift assays showing that ERF1 binds the promoter of *ATG8a*, *ATG8d* and *ATG13b* but not *ATG1a* and *ATG1b*. The experiments were repeated twice with the similar results.

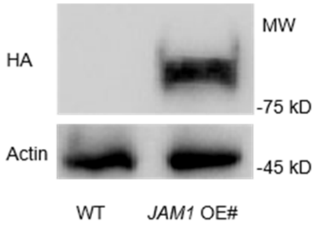

Supplementary Fig. 11 *JAM1*-overexpressing (*JAM1* OE#) transgenic tomato plants. Immunoblotting analysis of JAM1 protein in wild-type (WT, Ailsa Craig backgournd) and *JAM1* OE # plants using anti HA antibody. The Actin protein served as loading control.

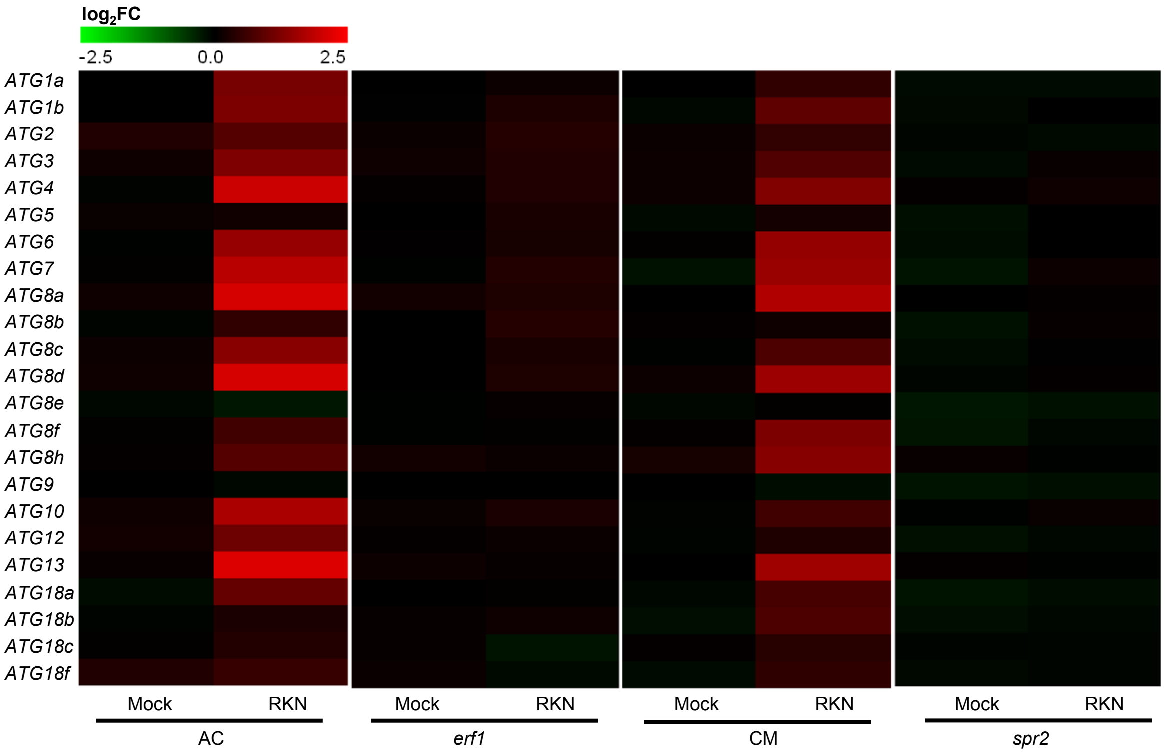

Supplementary Fig. 12 Heat-map of *ATGs* expression of Ailsa Craig (AC), *erf1*, Castlemart (CM) and *spr2* tomato roots with or without RKN infection. Transcript levels were determined using RT-qPCR and cluster analysis was performed by MeV version 4.9 and data were transformed by $\log_2$-fold change (FC). The color bar at the top shows expression levels. Data are presented as means of 3 biological replicates ± SD.
